# Gravity, intracranial pressure, and cerebral autoregulation

**DOI:** 10.14814/phy2.14039

**Published:** 2019-03-25

**Authors:** Lonnie G. Petersen, Shigehiko Ogoh

**Affiliations:** ^1^ Department of Orthopeadic Surgery University of California San Diego California; ^2^ Department of Biomedical Sciences University of Copenhagen Copenhagen Denmark; ^3^ Department of Biomedical Engineering Toyo University Tokyo Japan

## Abstract

Manipulating gravitational stress may affect cerebral autoregulation. These effects can help increase our understanding of the integrative cardiovascular‐ and cerebral physiology.

Gravity is a relentless fact of life on Earth. With each change in posture, the vector of all hydrostatic pressure gradients is altered, and regional pressures affected. Due to the elongated shape of the human body, our cardiovascular system is particularly sensitive and when upright gravity reduces cardiac output by remarkable 2 L/min. In response to the gravitational stress, an intricate system of baroreceptors and compensating blood pressure reflexes have evolved. The most important baroreceptors are located just below the brain and thus at an advantageous position to monitor and safeguard blood pressure and flow to the brain.

The cerebral perfusion pressure is defined as the pressure gradient across the brain and calculated by the difference between the arterial blood pressure at brain level and the intracranial pressure (ICP) (Petersen et al. 2016). To maintain perfusion pressure to the brain, elevations in ICP are counterbalanced by equal elevation in arterial blood pressure (Guild et al. 2018). The role of ICP is thus well‐recognized in pathology and any physician will cringe at the thought of what elevated ICP will do to cerebral perfusion, especially in the case of head‐trauma where cerebral autoregulation may be temporarily impaired or knocked out. Despite this, the role of ICP in normal everyday regulation of cerebral perfusion is largely unrecognized – this is in part because the pressure range of ICP has been believed to be very small compared to arterial blood pressure and therefore ignored, and perhaps moreover because of the invasive nature of ICP‐recordings and the limited available data on ICP variability in healthy humans. With this well‐written article, Stok et al. (2019) open up this important question of role of intracranial pressures and cerebrospinal fluid movement for cerebral autoregulation.

Cerebral autoregulation is a broad term that indicates the ability of the human brain to maintain appropriate blood flow despite changes in arterial blood pressure. A broad array of intrinsic mechanisms and systemic neural reflexes contribute to cerebral autoregulation. The term “static” cerebral autoregulation refers to the brains ability to maintain relatively constant flow within mean arterial blood pressure range of some 60–150 mmHg (Lassen 1974). The more recently coined term “dynamic” cerebral autoregulation describes the brain's ability to compensate for rapid changes in perfusion pressure. The buffering capacity of the cerebral vascular bed depends on the frequency of the fluctuation in perfusion pressure; high‐frequency fluctuations in arterial blood pressure are translated more directly to cerebral blood flow velocity, while slower changes are better dampened indicating more efficient autoregulation. Furthermore, a rise or fall in arterial partial pressure of CO_2_, which is a potent cerebral vasodialator, can increase or decrease cerebral blood flow independently of the autoregulation (Lennox and Gibbs 1932). Conversely, low arterial blood pressure alters the CO_2_ reactivity of the cerebral vasculature (Harper and Glass 1965) and a decrease or increase in CO_2_ partial pressures can improve or attenuate dynamic cerebral autoregulation (Aaslid et al. 1989). Thus, both direct and indirect effects of CO_2_ on the cerebral vasculature interfere with cerebral autoregulation adding to the complexity of the integrated physiology.

Daily fluctuates in ICP are determined by the sum of the volumes of intracranial arterial and venous pressure and the cerebrospinal fluid (CSF) pressure. The overall brain pressure is, so to say, the resultant balance of these three fluid columns. Within the rigid confinements of the skull, the second to second arterial inflow is, of course, perfectly matched by venous outflow and each pulse wave is furthermore buffered by CSF movements to and from the spinal cavity. The three fluid systems are thus synchronized and interact in a compensatory fashion.

Because of the eccentric placement of our brain at the very top of these fluid columns, the daily postural fluctuations in ICP are quite significant (Petersen et al. 2016). Traditionally, cerebral autoregulation is assessed by transfer function from systemic blood pressure to changes in cerebral perfusion. If the gravitational vector is kept constant, that is, body position is maintained, changes in blood pressure and ICP/cerebral perfusion pressure are congruent and this assessment holds true. However, during a change in posture, the arterial blood pressure at heart level is affected less than ICP and cerebral perfusion pressure because the hydrostatic indifference point is located close to heart level (Petersen et al. 2014). As dynamic changes in the gravitational vector affect regional arterial pressure and ICP/cerebral perfusion pressure differently, it is possible that dynamic cerebral autoregulation is over‐ or underestimated. Furthermore, compliance of the brain‐tissue and thus pressure‐wave propagation within the brain is affected by posture, which may in itself also affect cerebral autoregulation. In other words, ICP may affect cerebral autoregulation differently in upright versus supine postures and could be considered an independent modifying factor in dynamic regulation of cerebral blood flow.

Dr. Stok and colleagues (Stok et al. 2019) illustrate the complex interaction of gravitational fluid‐shifts, regional pressures, and respiration for cerebral autoregulation. Using both static and dynamic manipulation of the gravitational vector by whole‐body head‐up tilt and sinusoidal oscillations, responses in cerebral blood flow velocity and arterial blood pressure are reported. Further attempts to interpret data are done by mathematic modeling of CSF movements to and from the spinal canal. Despite the fact that no final conclusions can be drawn from the results, Stok et al. deserves much credit for the truly integrative approach to unravel the complex physiology behind cerebral autoregulation. The article raises the important question of the role of normal gravitational ICP fluctuations for cerebral autoregulation.

Changes in posture, whether static or dynamic, may be used to manipulate the magnitude and direction of the gravitational vector and the resultant hydrostatic pressure gradients. However, the only way to eliminate hydrostatic gradients altogether is by weightlessness which thus constitutes an important tool for investigating effects of gravitational stress. The indications of impaired intracranial pressure regulation in some astronauts during and following long‐term spaceflight (Lee et al. 2017) along with indications of possible impaired cerebral autoregulation (Blaber et al. 2011) begs further investigation of effects of gravity and weightlessness on cerebral perfusion and function.
